# Action of Ether Injected into the Arteries

**Published:** 1847-10

**Authors:** 


*Action of Ether injected into the Arteries.*—According to the experiments of M. Flourens, it would appear that ether, when injected into the blood-vessels, has an inverse effect to that observed after the inhalation of the fluid. When inhaled, ether suspends sensation before it interferes with the power of motion; but, when injected into the arteries, M. Flourens found that it destroyed the power of motion previously to exerting any influence on sensation: indeed, sensation remains unaffected, unless the quantity of ether employed is very large.—*Ibid, from Gaz. des Hôpitaux.*

